# Impact of electronic cigarette use on the increased risk of diabetes: the Korean Community Health Survey

**DOI:** 10.4178/epih.e2024029

**Published:** 2024-02-13

**Authors:** Wonseok Jeong, Seungju Kim

**Affiliations:** 1 Department of Public Health, Graduate School, Seoul National University, Seoul, Korea; 2 Department of Health System, College of Nursing, The Catholic University of Korea, Seoul, Korea

**Keywords:** Electronic cigarettes, Dual smoking, Diabetes, Conventional cigarettes

## Abstract

**OBJECTIVES:**

Only a few studies have solely investigated the health impacts of electronic cigarettes on diabetes while considering the impact of conventional cigarettes. Therefore, this study aimed to examine the effect of electronic cigarette smoking on diabetes in Korean dual smokers, electronic cigarette smokers, conventional cigarette smokers, and non-smokers.

**METHODS:**

Data were obtained from the 2021 and 2022 Korean Community Health Surveys of 460,603 Korean adults. The main independent variable was smoking behavior. Participants were categorized according to their smoking behavior, as dual smokers, electronic cigarette smokers, conventional cigarette smokers, and non-smokers. The dependent variable, the presence of diabetes, was defined by a doctor’s diagnosis. Multiple logistic regression analysis was performed to examine the association between smoking behavior and diabetes. Subgroup analyses were also performed to investigate the associations among different socioeconomic groups.

**RESULTS:**

Conventional cigarette smokers had a higher risk of diabetes than did non-smokers (adjusted odds ratio [aOR], 1.22; 95% confidence interval [CI], 1.19 to 1.26). More importantly, those who only vaped electronic cigarettes were at high risk of diabetes (aOR, 1.15; 95% CI, 1.01 to 1.31). Lastly, dual smokers had the highest aOR for diabetes among other smoking behavior groups (aOR, 1.39; 95% CI, 1.22 to 1.58). Dual smoking was associated with the highest risk of diabetes in most subgroups.

**CONCLUSIONS:**

This study suggests that conventional cigarette use and smoking electronic cigarettes negatively impact diabetes, and using both types leads to worse health outcomes. Therefore, cessation of all types of smoking is necessary for a healthy life.

## GRAPHICAL ABSTRACT

[Fig f1-epih-46-e2024029]

## Key Message

This study investigated how smoking electronic cigarettes affects diabetes among Korean dual smokers, electronic cigarette smokers, conventional cigarette smokers, and non-smokers. Data were obtained from the 2021 and 2022 Korean Community Health Surveys of 460,603 Korean adults. We found that using traditional cigarettes or electronic cigarettes adversely affects diabetes, and the combined use of both can lead to worse health outcomes. Therefore, quitting all forms of smoking is essential for maintaining a healthy life.

## INTRODUCTION

Diabetes is an increasing global health concern. According to the International Diabetes Federation Diabetes Atlas, the global prevalence of diabetes among adults aged between 20 years and 79 years steadily increased from 8.8% in 2017 to 9.3% in 2019 [1]. Moreover, it is estimated that there will be 783 million adults with diabetes worldwide if this trend continues [2]. Therefore, appropriate management through early detection is important to reduce the growing burden of the disease; however, early detection is difficult as diabetes slowly progresses without symptoms [3].

Previous studies have shown that more than half of the individuals with diabetes are unaware of their condition [4]. Various complications usually increase because of low awareness of the disease and delayed diagnosis, and the mortality risk of individuals with diabetes is known to be 2-3 times higher than that of the general population due to asymptomatic disease [5,6]. Therefore, identifying vulnerable groups through disease risk factor assessments and providing early intervention is important for the early detection and prevention of diabetes.

Various lifestyle factors, including eating habits, regular exercise, and smoking, are well-known risk factors for diabetes [5]. Particularly, smoking is not only a risk factor for various diseases, including diabetes [6,7], but can also lead to the early onset of various complications [8]. Smoking is known to alter endothelial function through direct toxic effects, which can lead to vascular phenotype dysfunction and loss of balance in the hemostatic system [9]. The negative effects of smoking on health were not recognized in Korea in the 1980s, despite the harmful effects of cigarettes, and the smoking rate was significantly high, with approximately 8 males of 10 males being smokers [10,11]. However, the negative health effects of smoking gained recognition with the improvement in income levels and increasing interest in health, and in conjunction with government regulatory policies, such as increasing cigarette prices, the smoking rate decreased from 66.3% in 1998 to 39.4% in 2015 [12]. Contrary to this trend, the use of electronic cigarettes, which are promoted as a healthy alternative to regular cigarettes and a smoking cessation aid, has actually increased [12,13]. Specifically, the electronic cigarette use rate rapidly increased from 2.0% in 2013 to 7.1% in 2015, and contrary to the original marketing goal, existing smokers are not switching to electronic cigarettes; instead, the number of dual users is increasing [14].

Many studies have revealed the relationship between conventional cigarette smoking and diabetes, leading to major changes in the smoking patterns of smokers [6]. Recently, a few studies reported the adverse health effects of dual smoking; however, they did not exclusively include the impact of electronic cigarettes [12,15]. Similarly, these studies scrutinized the health impact of electronic cigarettes and omitted that of conventional cigarettes [16]. Despite the increasing global health burden of diabetes and the use of electronic cigarettes, studies on the relationship between distinct electronic cigarette smoking and diabetes are limited. This research gap led us to investigate the potential connection between diabetes and smoking behaviors, including both electronic and conventional cigarettes as key indicators, considering the disparities in cigarette smoking prevalence among different demographic and socioeconomic status groups [8,17].

Therefore, this study aimed to examine the association between smoking behavior and diabetes in Korean adults, comparing those with and without diabetes among dual smokers, electronic cigarette smokers, conventional cigarette smokers, and non-smokers.

## MATERIALS AND METHODS

### Data and study participants

Data for this study were obtained from the 2021 and 2022 Korean Community Health Survey (KCHS), which has been collecting information on public health status and health behaviors at the community level since 2008. The KCHS is conducted by the Korean Centers for Disease Control and Prevention (KCDC, currently the Korea Disease Control and Prevention Agency) among adults aged ≥ 19 years via interviews from 255 communities in Korea. Sample areas were selected using stratified systematic sampling methods, and sample households were chosen employing a two-step systematic sampling technique to ensure that the sample units represent the entire population [18,19]. Details of the KCHS have been described elsewhere [20].

Among the 461,027 individuals who participated in the surveys between 2021 and 2022, 424 with missing covariates, such as smoking, alcohol consumption, and subjective health statuses, were excluded. Ultimately, the sample size was 460,603.

### Variables

The primary independent variable was smoking behavior. Participants were classified into the following four groups: dual smokers, electronic cigarette smokers, conventional cigarette smokers, and non-smokers. Individuals who answered “no” to the questions “Do you smoke a conventional cigarette now?” and “Do you vape an electronic cigarette now?” and “yes” to both questions were classified into the “non-smoker” and “dual smoker” groups, respectively. Individuals who answered “yes” to only one of the questions were categorized into the “conventional cigarette-alone smoker” or “electronic cigarette-alone smoker” group based on their respective answers.

The demographic characteristics included in the study were participants’ age (19-39, 40-59, and ≥ 60 years) and gender. Socioeconomic factors included the participants’ education level (middle school or lower, high school, and college or higher), region (urban or rural), and occupation. The region was categorized into urban and rural areas depending on whether it was a metropolitan city. The occupation categories followed the Korean version of the Standard Classification of Occupations, which was reclassified into four categories, as follows: white-collar (office work), pink-collar (sales and services), blue-collar (forestry, fishery, armed forces occupation, and agriculture), and unemployed [21].

Health-related characteristics included alcohol consumption status (heavy, moderate, or light consumption) according to the average number of days spent consuming alcohol (≥ 2, ≤ 1 day/wk, or 1 day/mo, respectively), regular exercise (yes or no), self-reported health status (good, normal, or bad), and body mass index (BMI; overweight or normal). Regular exercise was defined as 75 min/wk of vigorous aerobic exercise intensity or 150 min/wk of moderate aerobic intensity, following the World Health Organization (WHO) recommendation for physical activity [22]. A BMI of ≥ 23 kg/m^2^ was set as the cut-off point in this study, following the WHO definition of overweight in Asian adults [23].

Diabetes was included as the primary dependent variable. Diabetes status was defined depending on an answer to the question, “Have you ever been diagnosed with diabetes by a doctor?”

### Statistical analysis

Chi-square tests were used to analyze the study population’s general characteristics. Multiple logistic regression analysis was performed to examine the association between diabetes and smoking behavior after adjusting for potential confounding variables, including demographic, socioeconomic, and health-related characteristics. Finally, subgroup analyses were conducted to investigate the association between diabetes and smoking behaviors based on demographic and socioeconomic groups. The results are expressed as adjusted odds ratios (aORs) with 95% confidence intervals (CIs). Statistical significance was considered at p-value < 0.05, and all data analyses were conducted using the SAS version 9.4 (SAS Institute Inc., Cary, NC, USA).

### Ethics statement

The KCHS protocol was approved by the Institutional Review Board of KCDC (approval No. 2016-10-01-P-A). The requirement for informed consent was waived since the database was anonymized by confidentiality guidelines before distribution.

## RESULTS

Table 1 shows the study population’s general characteristics. Overall, only a few of the participants had diabetes (12.9%). Dual and electronic cigarette smokers represented 1.0% and 1.1% of the total participants, respectively. Additionally, 15.2% and 82.7% of the participants were conventional cigarette-alone smokers and non-smokers, respectively.

Table 2 shows the association between smoking behavior and diabetes prevalence among Korean adults, after adjusting for other confounding variables. Current conventional cigarette-alone smokers presented with a higher aOR for diabetes than did nonsmokers (conventional smokers: aOR, 1.22; 95% CI, 1.19 to 1.26). More importantly, individuals who only vaped electronic cigarettes were at a high risk of dyslipidemia, compared with nonsmokers (electronic cigarette smokers: aOR, 1.15; 95% CI, 1.01 to 1.31). Finally, individuals currently using conventional and electronic cigarettes were at the highest risk of diabetes (dual smokers: aOR, 1.39; 95% CI, 1.22 to 1.58). Individuals who exercised regularly had a lower risk of dyslipidemia compared with those who did not (yes: aOR, 0.97; 95% CI, 0.94 to 0.99). Good self-reported health status was also related to a lower risk of diabetes (good: aOR, 0.28; 95% CI, 0.28 to 0.29; normal: aOR, 0.55; 95% CI, 0.54 to 0.57). Notably, these results were significant. Finally, male participants had a higher risk of diabetes than did females (males: aOR, 1.56; 95% CI, 1.53 to 1.60).

Table 3 shows the results of the subgroup analyses between smoking behavior and diabetes based on age, sex, educational level, region, and occupational classification. In each smoking group, individuals with higher educational levels had the highest aORs, with statistical significance. Furthermore, the results for dual and electronic cigarette-alone smokers were solely significant among the most educated groups. Similarly, white-collar workers showed significant aORs in all smoking behavior groups. White-collar dual and electronic cigarette-alone smokers were at 1.54 and 1.29 times higher risks of diabetes, respectively, than were white-collar non-smokers. In summary, dual smokers had the highest risks of developing diabetes in most socioeconomic groups.

## DISCUSSION

Individuals frequently neglect the potential adverse effects of electronic cigarettes due to insufficient authoritative information about novel smoking devices. However, considering the negative health effects of conventional cigarettes, the potential health effects of electronic cigarettes cannot be overlooked. This study identified the health impacts of electronic cigarette smoking on the increased risk of diabetes. A strong correlation between dual smoking and diabetes was observed; dual smokers had the highest risk of developing diabetes among the different smoking behavior groups.

Although the relationship between smoking and diabetes remains unclear, several studies, including animal experiments, have suggested a correlation. In experimental studies, exposure to conventional and electronic cigarettes significantly increased insulin resistance, compared with non-exposure [24]. Similarly, observational studies in humans have shown that electronic cigarettes are associated with insulin resistance [25]. However, one study found that electronic cigarettes were not associated with insulin resistance in both humans and animals [26]. Although no clear mechanism for the relationship between smoking and diabetes has been proposed, these studies suggest that nicotine and other chemicals may increase diabetes due to inflammation, vascular effects, and insulin resistance [24,27,28]. Therefore, as conventional and electronic cigarettes can cause diabetes, smokers who use both products may have an increased risk of diabetes, compared with those who use a single product.

So far, the Korean government has implemented various policies that consider the negative health effects of conventional cigarettes [29], and particularly, the increase in cigarette retail prices has had a positive impact on increasing the smoking cessation rate [30]. However, because of scarce research on the adverse health efJfects of electronic cigarettes, the regulatory actions of the Korean government on electronic cigarettes are less restrictive than those on conventional cigarettes, leading to a surge in imports and sales of electronic cigarettes following the tax hike on conventional cigarettes [31]. Similarly, the use of electronic cigarettes among Korean adults has consistently increased due to the belief that it is safe [12]. Therefore, this presents a clear and advantageous course of action for the Korean government to take proactive steps in informing individuals with misconceptions never to start smoking electronic cigarettes and current users to quit.

Dual smokers presented the highest aOR for diabetes across the majority of subgroups. Additionally, the use of both types of cigarettes increased the risk of diabetes to the greatest extent among groups with different smoking behavior, regardless of age, region, and occupational classification, except for pink-collar workers. Special caution is warranted due to the highest aOR observed for dual smoking in the oldest age group, particularly when compared with that of dual smoking in other age groups. Moreover, considering the reporting bias from Korean female, statistically insignificant results among female and pink-collar workers are understandable [32]. Educational level further substantiates the notion that dual smoking and electronic cigarette use are negatively associated with the development of diabetes. According to a previous study, individuals with higher education levels had greater risks of using electronic cigarettes than did those with lower education levels [33]. Individuals with higher education levels tend to perceive electronic cigarettes as a more “sophisticated” alternative to tobacco products, and the insufficient scientific evidence on the negative health impact of electronic cigarettes likely serves as a strong incentive for educated people to hold such beliefs.

Our study has some limitations. First, the effect of the coronavirus disease pandemic on diabetes due to lifestyle changes was excluded from this investigation. Second, participants in the KCHS underwent face-to-face interviews, which helped build trust and rapport between the interviewers and respondents; however, it might have also led interviewees to refrain from reporting behaviors that were socially unacceptable. Third, the cause and effect, as well as the direction of the relationships, could not be observed because of the study’s cross-sectional design. Similarly, as changes in individual lifestyle habits cannot be evaluated, long-term research that considers these changes will be necessary. Lastly, this study could not consider individual smoking frequency due to data limitations; therefore, additional studies that consider these factors are necessary. Despite these limitations, the study had the following strengths. The KCHS is conducted by a national institution; therefore, the data obtained from it are more reliable and representative of the entire Korean population than those obtained by private institutions. Furthermore, this is one of the few studies to identify the different health impacts of electronic cigarettes while considering both dual and conventional smoking.

Many people have used electronic cigarettes since their introduction with the firm belief that they are safer than conventional cigarettes [34]. Furthermore, many smokers currently use both types of cigarettes, believing that smoking conventional cigarettes alone and using both types of cigarettes yield similar results. Because insufficient information exists regarding the health effects of smoking electronic cigarettes, our research identified the relationship between different smoking patterns and diabetes prevalence. We discovered that both electronic cigarette-alone and dual smokers were more likely to have diabetes, compared with non-smokers.

In conclusion, considering the increasing use of electronic cigarettes and the subsequent increase in the number of dual smokers, a full understanding of their associated risks is essential to prevent potential harm. Therefore, the results of this study can be used to inform people about different smoking patterns. Consistent with previous studies, individuals who smoked conventional cigarettes alone were encouraged to quit smoking to improve their health. Dual smokers should also quit smoking entirely or discontinue the use of one type of cigarette. Current conventional cigarettesalone smokers and non-smokers should never attempt to use electronic cigarettes, given the misconception that they are safe. Additionally, the results of this study provide scientific evidence for the Korean government to address diabetes management guidelines according to different socioeconomic and demographic groups. However, further studies are required to clarify the causal relationship between electronic cigarette smoking and diabetes.

## Figures and Tables

**Figure f1-epih-46-e2024029:**
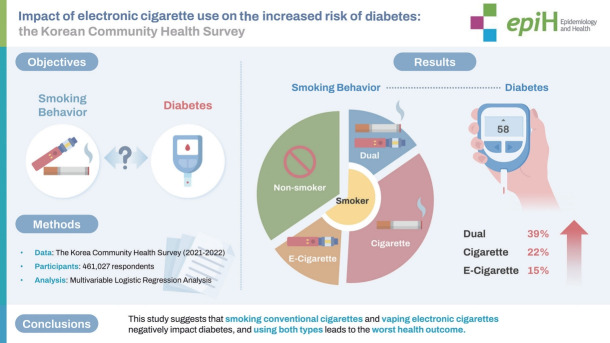


**Table 1.** General characteristics of the study population (n=460,603)

Characteristics	Total	Diabetes		p-value
		Yes	No	
Total	460,603 (100)	59,320 (12.9)	401,283 (87.1)	
Smoking behavior				<0.001
Dual smoker	4,787 (1.0)	277 (5.8)	4,510 (94.2)	
Conventional cigarette smoker	70,063 (15.2)	9,359 (13.4)	60,704 (86.6)	
Electronic cigarette smoker	5,027 (1.1)	276 (5.5)	4,751 (94.5)	
Non-smoker	380,726 (82.7)	49,408 (13.0)	331,318 (87.0)	
Sex				<0.001
Male	210,427 (45.7)	29,778 (14.2)	180,649 (85.8)	
Female	250,176 (54.3)	29,542 (11.8)	220,634 (88.2)	
Age (yr)				<0.001
19-39	97,968 (21.3)	1,201 (1.2)	96,767 (98.8)	
40-59	156,204 (33.9)	13,019 (8.3)	143,185 (91.7)	
≥60	206,431 (44.8)	45,100 (21.8)	161,331 (78.2)	
Educational level				<0.001
Middle school or lower	146,920 (31.9)	32,607 (22.2)	114,313 (77.8)	
High school	133,654 (29.0)	16,327 (12.2)	117,327 (87.8)	
College or higher	179,819 (39.0)	10,352 (5.8)	169,467 (94.2)	
Region				<0.001
Urban area	134,893 (29.3)	15,395 (11.4)	119,498 (88.6)	
Rural area	325,710 (70.7)	43,925 (13.5)	281,785 (86.5)	
Alcohol consumption status				
Heavy consumption	83,326 (18.1)	8,850 (10.6)	74,476 (89.4)	<0.001
Moderate consumption	187,750 (40.8)	16,375 (8.7)	171,375 (91.3)	
Light consumption	189,527 (41.1)	34,095 (18.0)	155,432 (82.0)	
Occupational classification				<0.001
White-collar	95,027 (20.6)	5,050 (5.3)	89,977 (94.7)	
Blue-collar	135,318 (29.4)	19,690 (14.6)	115,628 (85.4)	
Pink-collar	58,500 (12.7)	5,252 (9.0)	53,248 (91.0)	
None	171,758 (37.3)	29,328 (17.1)	142,430 (82.9)	
Self-reported health status				<0.001
Good	186,078 (40.4)	11,325 (6.1)	174,753 (93.9)	
Normal	192,708 (41.8)	25,290 (13.1)	167,418 (86.9)	
Bad	81,817 (17.8)	22,705 (27.8)	59,112 (72.2)	
Body mass index				<0.001
Overweight	250,126 (54.3)	38,104 (15.2)	212,022 (84.8)	
Normal	210,477 (45.7)	21,216 (10.1)	189,261 (89.9)	
Regular exercise				<0.001
Yes	91,885 (19.9)	9,572 (10.4)	82,313 (89.6)	
No	368,372 (80.0)	49,690 (13.5)	318,682 (86.5)	
Breakfast frequency (day/wk)				<0.001
5-7	305,951 (66.4)	50,916 (16.6)	255,035 (83.4)	
1-4	62,596 (13.6)	3,908 (6.2)	58,688 (93.8)	
0	92,056 (20.0)	4,496 (4.9)	87,560 (95.1)	
Year				<0.001
2021	229,007 (49.7)	28,447 (12.4)	200,560 (87.6)	
2022	231,596 (50.3)	30,873 (13.3)	200,723 (86.7)	

Values are presented as number (%).

**Table 2.** Factors associated with diabetes

Variables	Diabetes	p-value
Smoking behavior		
Dual smoker	1.39 (1.22, 1.58)	<0.001
Conventional cigarette smoker	1.22 (1.19, 1.26)	<0.001
Electronic cigarette smoker	1.15 (1.01, 1.31)	0.032
Non-smoker	1.00 (reference)	
Sex		
Male	1.56 (1.53, 1.60)	<0.001
Female	1.00 (reference)	
Age (yr)		
19-39	0.10 (0.09, 0.10)	<0.001
40-59	0.52 (0.51, 0.53)	<0.001
≥60	1.00 (reference)	
Educational level		
Middle school or lower	1.28 (1.24, 1.32)	<0.001
High school	1.20 (1.16, 1.23)	<0.001
College or higher	1.00 (reference)	
Region		
Urban area	1.04 (1.02, 1.07)	<0.001
Rural area	1.00 (reference)	
Alcohol consumption status		
Heavy consumption	0.76 (0.74, 0.78)	<0.001
Moderate consumption	0.83 (0.81, 0.85)	<0.001
Light consumption	1.00 (reference)	
Occupational classification		
White-collar	0.84 (0.81, 0.87)	<0.001
Blue-collar	0.90 (0.88, 0.92)	<0.001
Pink-collar	0.93 (0.90, 0.97)	<0.001
None	1.00 (reference)	
Self-reported health status		
Good	0.28 (0.28, 0.29)	<0.001
Normal	0.55 (0.54, 0.57)	<0.001
Bad	1.00 (reference)	
Body mass index		
Normal	0.59 (0.57, 0.60)	<0.001
Overweight	1.00 (reference)	
Regular exercise		
Yes	0.97 (0.94, 0.99)	0.010
No	1.00 (reference)	
Breakfast frequency (day/wk)		
5-7	1.59 (1.53, 1.64)	<0.001
1-4	1.12 (1.07, 1.18)	<0.001
0	1.00 (reference)	

Values are presented as adjusted odds ratio (95% confidence interval).

**Table 3.** Subgroup analysis of odds ratio for diabetes stratified by smoking behavior

Variables	Dual smoker	Conventional cigarette smoker	Electronic cigarette smoker	Non-smoker
Age (yr)				
19-39	1.42 (1.08, 1.88)	1.46 (1.25, 1.70)^*^	1.13 (0.81, 1.60)	1.00 (reference)
40-59	1.34 (1.13, 1.58)	1.24 (1.18, 1.30)^*^	1.17 (1.00, 1.38)^*^	1.00 (reference)
≥60	1.59 (1.09, 2.32)	1.14 (1.10, 1.18)^*^	1.29 (0.95, 1.75)	1.00 (reference)
Sex				
Male	1.30 (1.13, 1.48)	1.16 (1.13, 1.20)^*^	1.03 (0.90, 1.18)	1.00 (reference)
Female	1.45 (0.90, 2.35)	1.53 (1.41, 1.65)^*^	1.47 (1.00, 2.18)	1.00 (reference)
Educational level				
Middle school or lower	1.27 (0.77, 2.11)	1.13 (1.08, 1.18)^*^	1.27 (0.73, 2.20)	1.00 (reference)
High school	1.17 (0.94, 1.45)	1.13 (1.08, 1.19)^*^	1.02 (0.81, 1.28)	1.00 (reference)
College or higher	1.46 (1.23, 1.74)	1.38 (1.30, 1.18)^*^	1.20 (1.02, 1.42)^*^	1.00 (reference)
Region				
Urban area	1.56 (1.26, 1.93)	1.26 (1.19, 1.33)^*^	1.12 (0.90, 1.39)	1.00 (reference)
Rural area	1.31 (1.11, 1.54)	1.20 (1.16, 1.24)^*^	1.17 (1.00, 1.37)	1.00 (reference)
Occupational classification				
White-collar	1.54 (1.24, 1.91)	1.34 (1.24, 1.45)^*^	1.29 (1.05, 1.58)^*^	1.00 (reference)
Blue-collar	1.25 (1.01, 1.55)	1.07 (1.03, 1.12)^*^	0.86 (0.66, 1.12)	1.00 (reference)
Pink-collar	0.94 (0.65, 1.36)	1.22 (1.11, 1.34)^*^	0.96 (0.69, 1.33)	1.00 (reference)
None	1.45 (1.03, 2.04)	1.31 (1.25, 1.38)^*^	1.36 (1.00, 1.85)^*^	1.00 (reference)

Values are presented as adjusted odds ratio (95% confidence interval).

* p<0.05.
